# Platypnea–orthodeoxia syndrome in a postoperative patient: a case report

**DOI:** 10.1186/s13256-021-03185-7

**Published:** 2021-12-19

**Authors:** João Pinto Pereira, Benoit Ghaye, Pierre-François Laterre, Philippe Hantson

**Affiliations:** 1 Department of Intensive Care, Cliniques St-Luc, Université catholique de Louvain, Avenue Hippocrate, 10, 1200 Brussels, Belgium; 2 Department of Radiology, Cliniques St-Luc, Université catholique de Louvain, Brussels, Belgium

**Keywords:** Platypnea, Orthodeoxia, Pleural effusion, Pulmonary embolism, Partial anomalous pulmonary venous return

## Abstract

**Background:**

We report a case of platypnea–orthodeoxia syndrome observed in a complex clinical situation associating a bilateral pleural effusion, lobar pulmonary embolism, and a partial anomalous pulmonary venous return.

**Case presentation:**

A 57-year-old Caucasian woman developed acute dyspnea in the postoperative course of an elective gynecological surgery for advanced stage ovarian cancer. Preoperative evaluation had failed to reveal any respiratory or cardiac problem. After evidence of a low arterial oxygen saturation, blood gas analysis from the central venous line correctly inserted in the right internal jugular vein revealed a higher oxygen saturation than in the arterial compartment. A thoracic computed tomography showed bilateral pleural effusion, lobar pulmonary embolism, and a drainage of a left pulmonary vein into the left innominate vein. This unique combination resulted in an uncommon cause of platypnea–orthodeoxia syndrome.

**Conclusion:**

Often associated with right-to-left shunting, platypnea–orthodeoxia syndrome may be observed in complex clinical conditions with several factors influencing the ventilation/perfusion ratio. The paradoxical finding of a higher oxygen saturation in a central venous line than in an arterial line should prompt the clinician to look at the possibility of partial anomalous pulmonary venous return. No specific treatment is required in asymptomatic adults, except for an echocardiographic follow-up to detect the onset of pulmonary hypertension.

## Background

While postoperative dyspnea is a common symptom following major abdominal surgery, platypnea–orthodeoxia syndrome is rare and might correspond to numerous etiologies [1]. Chest x-ray examination and arterial blood gas analysis are first-line tests aiming to exclude the most common pulmonary or cardiac causes. Significant information can also be obtained by looking at the arteriovenous difference in oxygen (O_2_), with blood samples obtained from an arterial and a central venous line (CVL). The finding of a low arterial O_2_ saturation together with a high O_2_ venous saturation is quite unusual and should prompt further investigations.

## Case presentation

**Fig. 1:**
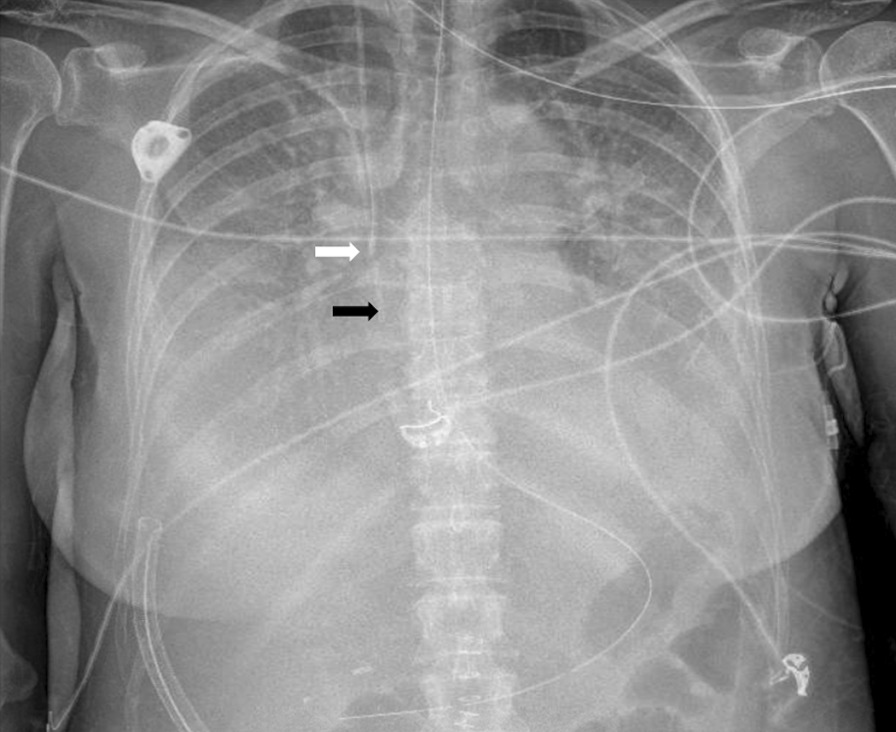
Chest x-ray examination showing the correct position of the tip of the Port-a-Cath (white arrow) and central venous (black arrow) catheter in the left internal jugular vein. Large bilateral pleural effusions are seen.

A 57-year-old Caucasian woman was recently diagnosed with advanced stage ovarian cancer. Otherwise healthy, she had initially consulted her general practitioner simply for abdominal distension. Further investigations [abdominal computed tomography (CT), serum CA125determination and laparoscopy] revealed a peritoneal carcinomatosis. The diagnosis of stage III (FIGO classification) ovarian cancer was established and the patient received a carboplatin-paclitaxel based chemotherapy regimen in a neoadjuvant setting. The indication of a debulking surgery with hyperthermic intraperitoneal chemotherapy (HIPEC) was retained by our institutional tumor board. A CVL was inserted via the right jugular vein into the superior vena cava in prevision of surgery and the appropriate positioning of the catheter was verified by chest x-ray. An implantable central venous catheter (Port-a-Cath) was already in place (Fig. 1).

The first 48 postoperative hours were marked by difficult pain management, hypotension, and transient hyperlactatemia responding to fluid replacement and norepinephrine. On postoperative day 3, she presented acute onset dyspnea when transferred from the bed to a chair, and a major drop in pulse oxygen saturation (from SpO_2_ 96% to 83%) justified the administration of oxygen (5 L/min) via a nasal cannula. On physical examination, her body temperature was 37.4 °C, blood pressure 135/81 mmHg, heart rate 122 beats/min, respiratory rate 20/min. No chest pain was reported. Pulmonary examination revealed a bilateral reduction of basal breath sounds with dullness at percussion. Chest x-ray examination (Fig. 1) showed bilateral pleural effusions that could largely explain patient’s dyspnea and oxygen desaturation. After switching from a nasal cannula to a nonrebreather mask (FiO_2_ 0.40), the patient’s condition seemed to stabilize in supine position. A measurement of the arteriovenous oxygen difference was obtained via sampling through the arterial and central venous line after the patient was again lying in supine position (Table 1).

**Table 1:** Comparison of arterial blood gas analysis from the central venous and arterial line in supine position

	Central venous line (superior vena cava)	Arterial line (left radial)
pH	7.44	7.47
Oxygen saturation (%)	94	91.5
Partial pressure O_2_ (mmHg)	66.6	57.9
Partial pressure CO_2_ (mmHg)	35.5	32.7
Fraction of inspired O_2_ (%)	40	40

Alveolar-arterial gradient O_2_: (150 − 1.25 × 32.7) − 57.9 = 51.25

**Fig. 2:**
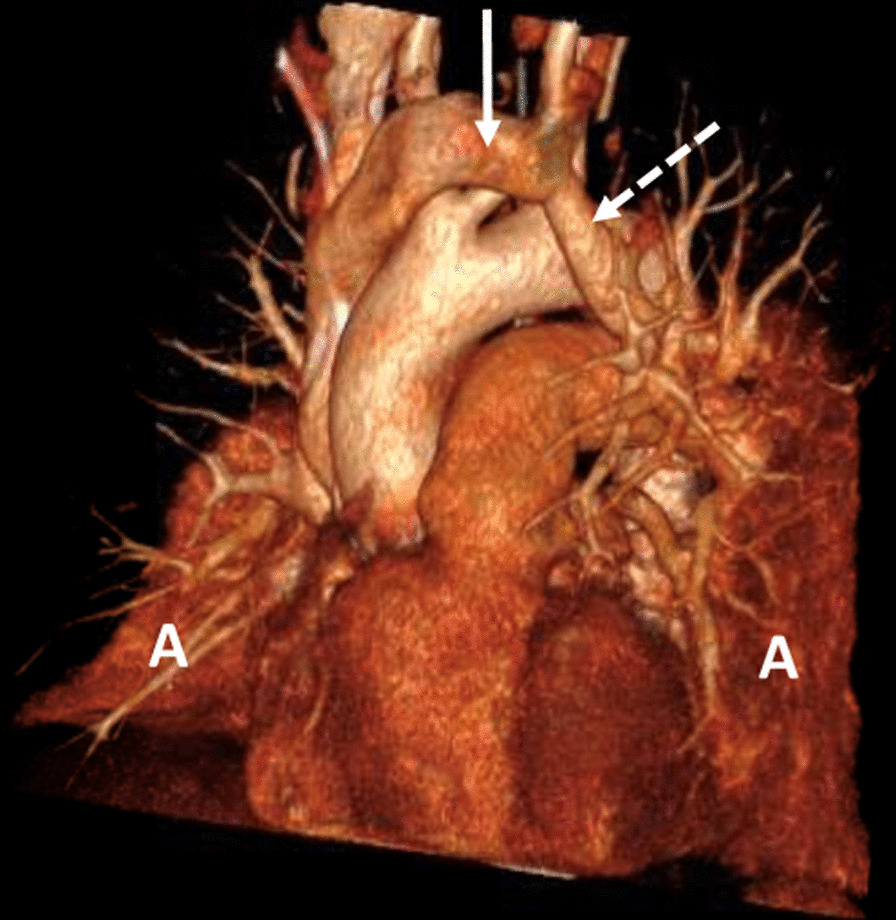
Left anterior oblique view of a three-dimensional reconstruction of CT pulmonary angiography. Full arrow: left innominate vein. Dotted arrow: left upper pulmonary vein showing an upwards course to drain into the left innominate vein. Large passive atelectasis (**A**) of both lower lobes is demonstrated.

A sampling error or a wrong positioning of the recent CVL was suspected, but a sample taken from the Port-a-Cath confirmed the venous value. A transthoracic echocardiography (TTE) with bubble test failed to show a right-to-left shunt, atrial septum was intact. No pulmonary hypertension was detected and right ventricle was not dilated. A CT pulmonary angiography (Fig. 2) showed large bilateral pleural effusions with atelectasis of both lower lobes, acute pulmonary embolism in the right middle and upper lobe pulmonary arteries, and ultimately an anatomical variant of the left upper pulmonary vein draining into the left innominate vein. Following bilateral chest drainage, the patient was treated with low molecular weight heparin twice daily. There was no evidence for deep venous thrombosis. This was followed by a significant clinical improvement, a disappearance of the platypnea–orthodeoxia complaints and a reduction of oxygen requirement over the following days.

At 1-year follow-up, TTE showed no right ventricular dilation and absence of pulmonary hypertension. From an oncological perspective, stability was also observed during her niraparib maintenance therapy, with absence of new lesions at a 1-year follow-up abdominal CT.

## Discussion and conclusions

Platypnea–orthodeoxia syndrome (P-OS) is an uncommon clinical manifestation that consists of breathlessness that aggravates in the upright position and relieves in recumbent position. Since its description by Burchell *et al*. in 1949, a vast majority of the reported P-OS seems to be linked to a right-to-left circulatory shunt, usually by means of a cardiac anatomic defect such as a patent foramen ovale (PFO) [1]. However, in some rare instances, P-OS seems to be solely associated to a ventilation/perfusion (V/Q) ratio mismatch. This could occur in patients with a history of chronic lung disease such as severe obstructive lung disease, postpneumonectomy and pulmonary vascular shunting [2, 3].

**Fig. 3:**
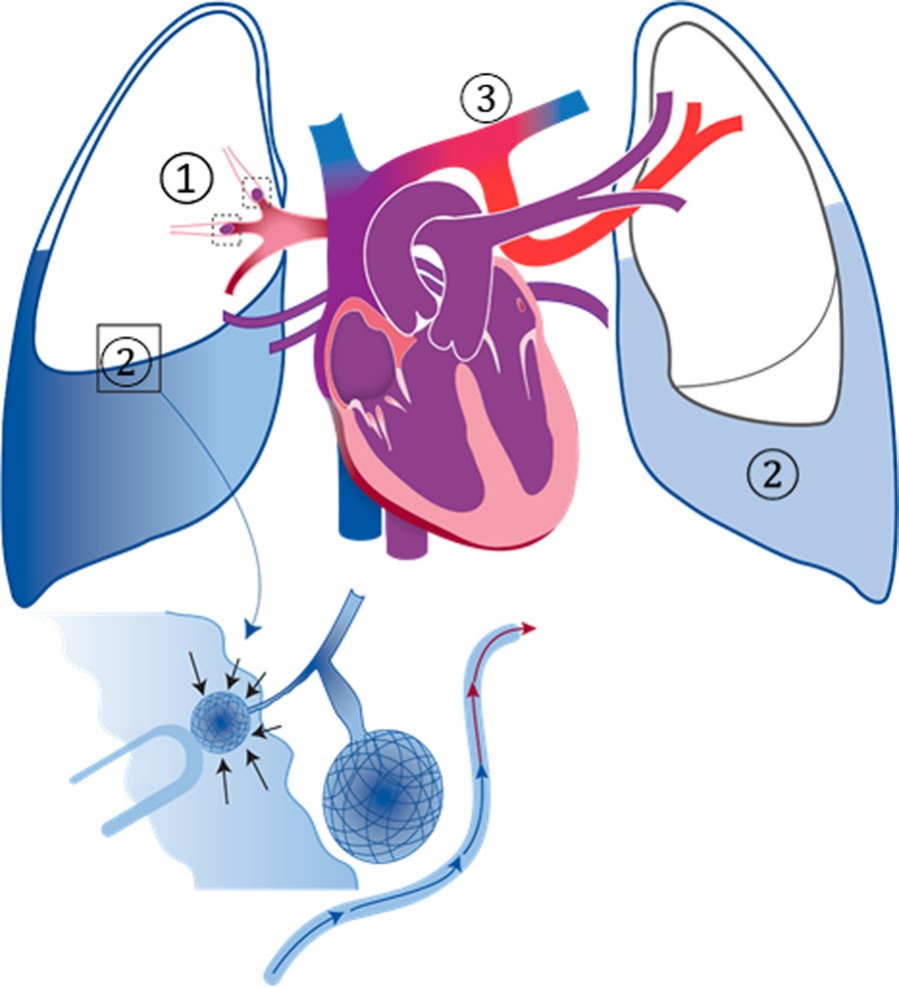
Possible mechanism for ventilation/perfusion (V/Q) ratio mismatch leading to PO-S. ① Pulmonary embolism ② Pleural effusion with passive atelectasis ③ Partial anomalous pulmonary venous return

In the present observation, several factors might have contributed to a V/Q mismatch (Fig. 3).

First, there was a documented acute pulmonary embolism (PE) involving the right middle and upper lobes. In the few reported cases of PO-S following PE, the presence of an intracardiac shunt was usually demonstrated [4–6]. In patients with PE and PFO, right-to-left shunting is very likely related to the acute increase in pulmonary artery pressure that determines the reversal of the shunt direction or the development of a right-to-left shunt while sitting upright, in the absence of a left-to-right shunt in a supine position. In the clinical observation reported by Brenner *et al*., however, no evidence of intracardiac shunt was documented in a 56-year-old man who presented with PO-S after bilateral lower-lobe pulmonary emboli [7]. As in our observation, the patient was investigated by TTE and CT with angiography, and the possibility of a transient PFO could not be excluded.

Second, the patient presented with major bilateral pleural effusion with passive atelectasis of both lower lobes. This would have resulted in significant V/Q mismatch due to hypoventilation of normally perfused segments. More pronounced anatomical or functional intrapulmonary shunting in the lower parts combined with a reduced potential for compensation in the upper parts of the lungs will then result in significant deoxygenation. Furthermore, in the present case, perfusion was reduced in the right middle and upper lobe following PE.

Third, the thoracic CT demonstrated a partial anomalous pulmonary venous return (PAPVR) without associated septal defect. The diagnosis of PAVPR is often made incidentally or postmortem, as most adult patients will remain asymptomatic [8]. Occasionally, it can be obtained by the chest x-ray examination showing a malposition of a recently inserted CVL, particularly when CVL was supposed to be inserted in the left internal jugular vein [9]. In the present observation, both Port-a-Cath and CVL catheter were correctly inserted in the right internal jugular vein. As in other previously published cases, the simultaneous determination of the arterial blood gas from the CVL and arterial line was helpful as the partial pressure in oxygen was paradoxically higher from the CVL compared with the arterial line [10–13]. This precluded the correct interpretation of the arteriovenous difference in oxygen that is usually extremely helpful for the management of a critically ill patient. Logically, PAPVR is associated with a left-to-right shunting effect but not with PO-S. There was here no evidence of elevated pulmonary pressure and subsequent right-to-left shunting through a PFO. The exact influence of the change from supine to upright position on the V/Q ratio of the physiologically preserved left upper lobe is not known. There is no objective reason for a worsening of PO-S.

In conclusion, while PO-S is often associated with right-to-left shunting, it may be observed in complex clinical conditions with several factors influencing the V/Q ratio. The paradoxical finding of a higher oxygen saturation in a CVL than in an arterial line should prompt the clinician to look at the possibility of PAPVR. No specific treatment is required in asymptomatic adults, except for an echocardiographic follow-up to detect the onset of pulmonary hypertension [14, 15].

## Data Availability

Not applicable.

## Abbreviations

O_2_: Oxygen
CVL: Central venous line
CT: Computed tomography
V/Q ratio: Ventilation/perfusion ratio
HIPEC: Hyperthermic intraperitoneal chemotherapy
TTE: Transthoracic echocardiography
PFO: Patent foramen ovale
PAPVR: Partial anomalous pulmonary venous return
